# Clinical characteristics and outcomes of patients with chondroblastoma undergoing surgery with various adjuvant procedures: a retrospective study of 59 cases

**DOI:** 10.1186/s12893-025-02782-3

**Published:** 2025-01-24

**Authors:** Toru Hirozane, Tetsuya Sekita, Eisuke Kobayashi, Tomoaki Mori, Naofumi Asano, Toru Udaka, Takashi Tajima, Rumi Nakagawa, Kazutaka Kikuta, Akira Yoshiyama, Hideo Morioka, Itsuo Watanabe, Ukei Anazawa, Michiro Susa, Keisuke Horiuchi, Yoshihisa Suzuki, Takeshi Morii, Robert Nakayama

**Affiliations:** 1 Department of Orthopaedic Surgery, Keio University School of Medicine, Shinjuku-Ku, Tokyo, Japan; 2 Department of Musculoskeletal Oncology, National Cancer Center Hospital, Chuo-Ku, Tokyo, Japan; 3 Department of Orthopaedic Surgery, Faculty of Medicine, Kyorin University, Mitaka, Tokyo, 181-8611 Japan; 4 Division of Musculoskeletal Oncology and Orthopaedic Surgery, Tochigi Cancer Center, Utsunomiya, Tochigi, Japan; 5 Department of Orthopaedic Surgery, National Hospital Organization Tokyo Medical Center, Meguro-Ku, Tokyo, Japan; 6 Department of Orthopaedic Surgery, Tokyo Dental College Ichikawa General Hospital, Ichikawa, Chiba Japan; 7 Department of Orthopaedic Surgery, National Defense Medical College, Tokorozawa, Saitama Japan; 8 Department of Orthopaedic Surgery, Tachikawa Hospital, Tachikawa-Shi, Tokyo, Japan

**Keywords:** Chondroblastoma, Bone neoplasm, Curettage, Local recurrence, Denosumab

## Abstract

**Background:**

Chondroblastoma is classified as a benign bone tumor. However, postoperative local recurrence remains a concern. We analyzed the factors contributing to chondroblastoma local recurrence and the clinical challenges associated with treating these patients.

**Methods:**

This retrospective study examined 59 patients followed up at our hospitals for ≥ 1 year after surgery during 1990–2020. The most common lesion site was the epiphyses of long bones (42 cases, 71%), including the femur, tibia, and humerus. Curettage was performed in 57 cases; 2 cases with an iliac lesion underwent resection. The median postoperative follow-up period was 47 months. Clinical features of chondroblastoma were retrospectively investigated, and local recurrence and postoperative functional outcomes were assessed.

**Results:**

Local recurrence occurred in 9% (5/57) of patients after curettage but not in the resected cases. The median time to local recurrence was 14 months. The local recurrence-free survival (LRFS) rate for all patients was 92.7% at 2 years and 88.3% at 5 years. All patients with local recurrence were aged < 17 years at the time of surgery. Local recurrence was observed in the proximal humerus in two cases and the calcaneus, acetabulum, and distal femur in one case each. None of the adjuvant procedures (high-speed burr, ablation, bone replacement materials, and preoperative denosumab) helped reduce local recurrence risk (*P* > 0.05). Trends toward fewer local recurrences were observed in the group treated using the high-speed burr and in the group not treated using bone replacement materials. Among the groups treated with bone replacement materials, artificial bone achieved the best LRFS rate, followed by allograft and autograft. At the final follow-up, the mean Musculoskeletal Tumor Society score was 29.8 (range: 25–30), indicating excellent postoperative functional outcomes. Joint degeneration was observed in five patients. Patients with local recurrence had a high degree of disability and joint deformity (*P* < 0.05). Two patients received preoperative denosumab and neither experienced local recurrence nor functional impairments.

**Conclusions:**

Good oncological and functional outcomes were achieved. Age < 17 years was associated with a high risk of local recurrence after curettage (*P* = 0.0198). Patients with local recurrence exhibited poorer functional outcomes. High-speed burr may help reduce the recurrence risk. If bone grafts are necessary, materials with low biocompatibility, including artificial bone, may be optimal. Managing patients with chondroblastoma should encompass curative and functional aspects.

**Supplementary Information:**

The online version contains supplementary material available at 10.1186/s12893-025-02782-3.

## Background

Chondroblastoma is a relatively rare benign bone tumor first described by Jaffe and Lichtenstein in 1942 [1]. Although metastasis and malignant transformation cases have been documented, they are extremely rare [2–5]. It primarily affects males in their teens to twenties and typically occurs in the epiphysis or apophysis of long bones [6]. The primary symptoms include pain, swelling, and claudication [7–10]. The pain is usually mild; however, there is often a prolonged course of symptoms. Joint effusions have been documented in some patients [6].

Histologically, chondroblastoma comprises stromal cells and multinucleated giant cells. The differential diagnosis includes primary aneurysmal bone cyst (ABC), giant cell tumor of bone (GCT), clear cell chondrosarcoma, chondromyxoid fibroma, and chondroblastoma-like osteosarcoma [11, 12]. A previous study showed that the G34W mutation in the histone-encoding *H3F3A* gene was present in 92% of patients with GCT, and the K36M mutation in the *H3F3A* or *H3F3B* gene was present in 95% of patients with chondroblastoma [13]. These mutations are found in the stromal cells and are mutually exclusive. The discovery of these mutations has allowed a definitive diagnosis of these tumors by genomic analysis [14]. Furthermore, developing antibodies that detect these single amino acid mutations has allowed for more accurate diagnosis by immunostaining [15, 16].

The treatment of choice for chondroblastoma is curettage, and few reports have documented cases treated with resection [6, 8, 9, 17–19]. Reportedly, the local recurrence rate after curettage is approximately 10% [17, 19]. Previous studies have shown the potential association of tumor location with an increased risk of local recurrence; the proximal humerus [6, 8, 9, 18, 20–23], proximal femur [8, 18–21, 23, 24], tarsus [8, 20, 23], and pelvis [9, 17, 18, 24] being the sites most frequently associated with local recurrence. The time from surgery to local recurrence varies among studies, with some suggesting it may occur less than 1 year after surgery [21, 25]. In contrast, others have reported that it may occur more than 4 years after surgery [9, 17, 18]. Risk factors for local recurrence other than the site of origin include preoperative radiologic findings (ABC-like changes) [18, 20], tumor size [7], age at surgery [18, 20], status of the epiphyseal line [21], time to symptom onset [11], and differences in surgical methods, such as the use of a high-speed burr [9]. Several studies have reported using denosumab, a humanized monoclonal antibody against RANKL [2, 26–28]; however, denosumab has not been established as a treatment option for chondroblastoma. Furthermore, due to the rarity of this condition, the clinical presentation of this entity, the risk factors for local recurrence, and the effectiveness of various adjuvant procedures have not yet been fully elucidated.

In this study, we retrospectively evaluated chondroblastoma patients who underwent surgery and sought to better understand the potential factors contributing to local recurrence and the clinical problems associated with treating these patients.

## Methods

**Fig. 1:**
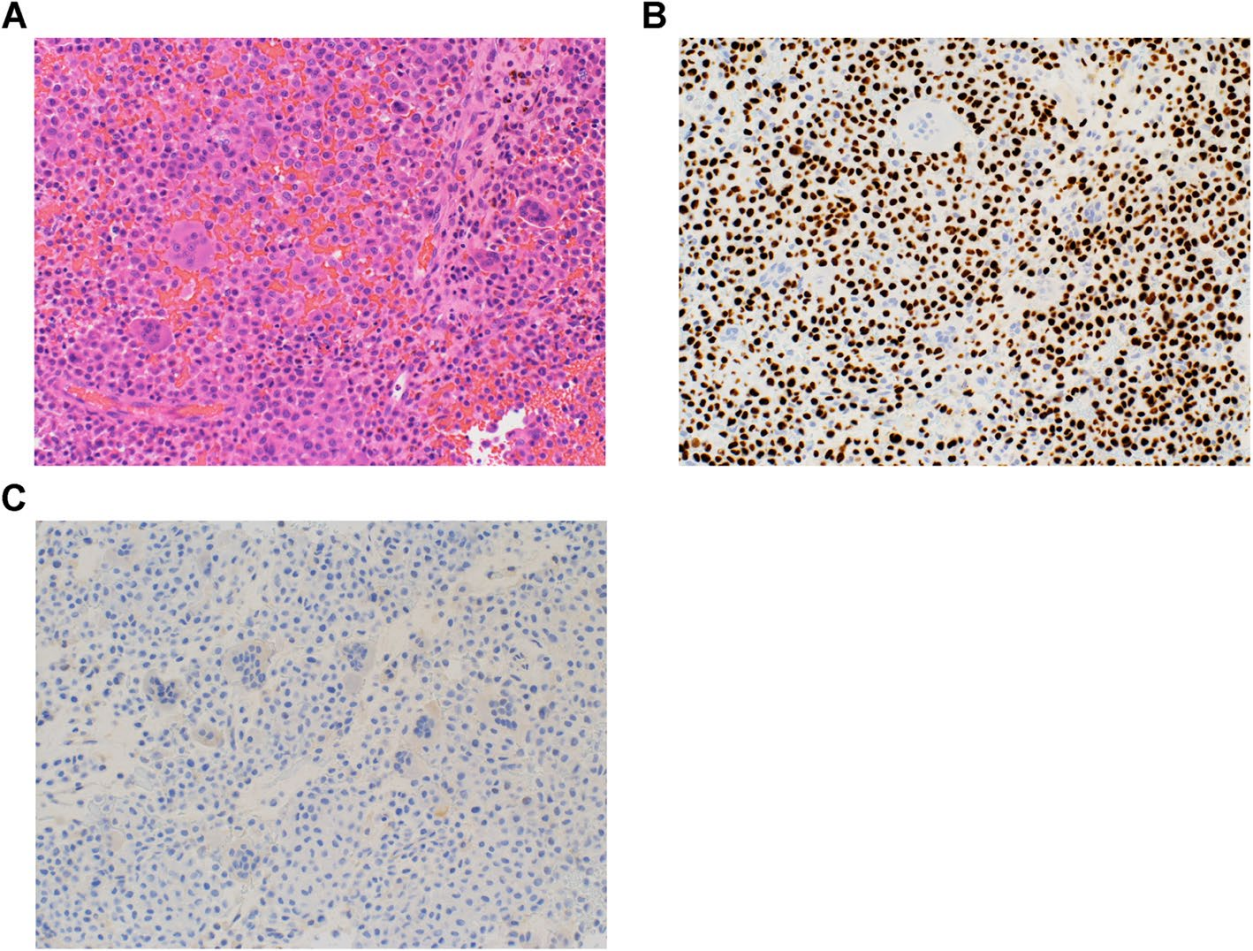
Pathological findings of chondroblastoma (40 ×). **A** H & E staining. **B** H3K36M immunostaining. **C** H3G34W immunostaining. H3K36M shows diffuse nuclear expression in the chondroblastic cells

This multicenter retrospective study was conducted at Keio University Hospital and its affiliated facilities. Patients diagnosed with chondroblastoma and treated between 1990 and 2020 were included in this study. Cases with more than one year of postoperative follow-up and accessible, detailed clinical information were included. Cases in which a definitive diagnosis of chondroblastoma was not possible due to pathological diagnostic challenges were excluded. Orthopedic surgeons involved in the treatment of musculoskeletal tumors at each hospital retrospectively collected and compiled clinical information. The study proposal was reviewed and approved by the Ethical Review Committee of each participating institution. Age, sex, site of origin, tumor size, local pain, range of motion of the affected joint, diagnosis of preoperative biopsy, magnetic resonance imaging (MRI) findings (especially ABC-like changes), epiphyseal line closure, pathological findings and H3K36M immunoreactivity (Fig. 1A-C) were evaluated.

Regarding surgery, we reviewed the type of surgery (curettage or en bloc resection), adjuvant surgical procedures (such as the use of a high-speed burr and thermal ablation by electrocautery), and bone grafting. The attending physician determined the presence of local recurrence based on imaging and clinical findings, and it was subsequently confirmed histologically by certified bone and soft tissue pathologists. The time of local recurrence was defined as the point when the attending physician diagnosed local recurrence based on imaging findings (primarily plain X-rays, and in some cases, CT or MRI) and clinical symptoms such as progressive pain. Cases subsequently confirmed as local recurrence through histological examination after surgery were classified as local recurrent cases. At the final visit, each attending physician assessed joint degeneration and the Musculoskeletal Tumor Society (MSTS) scores.

Descriptive statistics were used to present the demographic data. The Kaplan–Meier method determined the local recurrence-free survival (LRFS) rate. Differences in survival were assessed using the log-rank (Mantel–Cox) test. Since categorical variables with five or fewer cases were included in each table, Fisher’s exact test was employed to compare these variables. Continuous data, including age and follow-up period, were compared using the Mann–Whitney *U* test. Given the limited number of cases with local recurrence and the presence of outliers in each study item, we concluded that normality could not be assumed and thus employed this method instead of the t-test for the analysis. All the tests were two-tailed. *P* values < 0.05 were considered statistically significant. All statistical analyses were performed using GraphPad Prism 10 (GraphPad Software, CA, USA).

## Results

A total of 74 patients were enrolled in this study. Of these, 59 patients with detailed medical records and at least 1 year of follow-up after the initial surgery were evaluated.

### Patient background

**Fig. 2:**
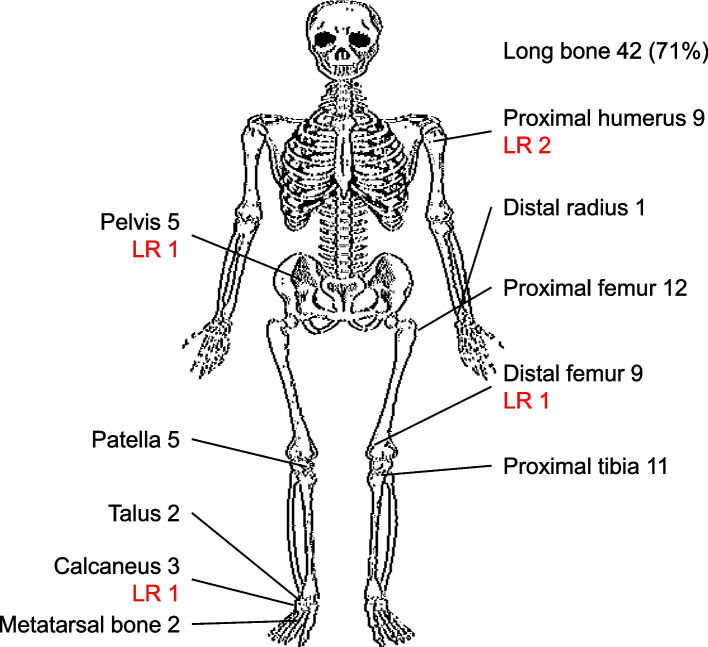
Locations of chondroblastomas. The site distribution of cases is shown. LR = Local recurrence. The most common locations include the proximal femur, proximal tibia, distal femur, and proximal humerus

The median age at the initial visit was 16 years, with a range of 8–42 years. This study included 42 male and 17 female patients. At the initial examination, pain and limited range of motion were evident in 38 (64%) and 15 (25%) patients. The median tumor size was 3 (1–10) cm. Nineteen patients (32%) exhibited ABC-like changes on the preoperative MRI. The most common tumor site was the long bones in 42 patients (71%). The distribution of tumor location and local recurrence is presented in Fig. 2. Biopsies were conducted on 29 patients (49%), revealing an initial histologic diagnosis of chondroblastoma in 24 cases (24/29, 83%), GCT in two cases (2/29, 7%), uncertain giant cell lesions in two cases (7%), and ABC in one case (3%). Cases initially diagnosed as ABC and GCT at the time of biopsy were later found to be positive for H3K36M immunostaining, leading to a revised diagnosis of chondroblastoma, and were thus included in this study. All tumor diagnoses were confirmed as chondroblastoma by surgical pathologists with expertise in bone and soft tissue pathology and were deliberated upon in a multidisciplinary tumor board.

### Clinical outcomes

**Fig. 3:**
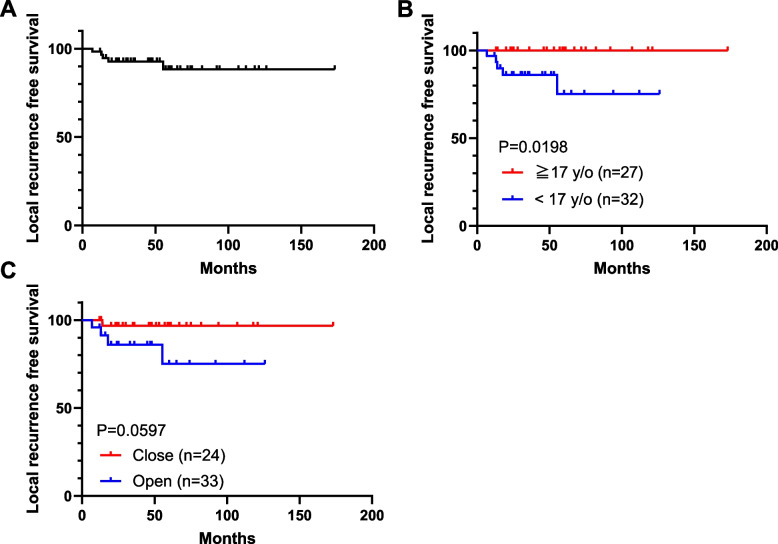
Local recurrence-free survival (LRFS). Survival curves, as determined by Kaplan–Meier analysis, are shown. **A** LRFS curve of all cases. **B** LRFS curves of the groups defined by age (< 17 years vs. ≧ 17 years) reveal a significant difference with a *P* = 0.0198 based on the log-rank test. **C** LRFS curves of the groups are defined by the status of the epiphyseal line (*P* = 0.0597)

**Fig. 4:**
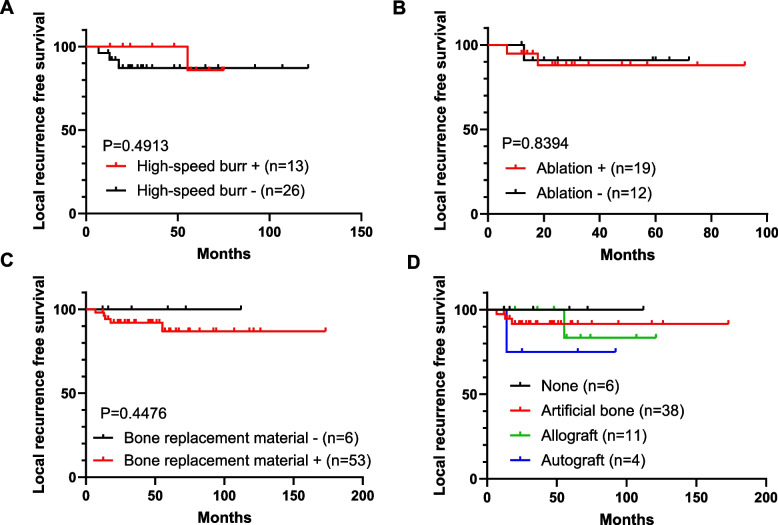
LRFS curves of the groups defined based on various adjuvant surgical procedures. LRFS curves of the groups defined by the usage of high-speed burr (**A)**, ablation (**B**), bone replacement materials (**C**), and the type of bone replacement materials (**D**)

Surgical procedures were curettage in 57 cases and resection in 2 cases (both involving the iliac bone). The median postoperative follow-up was 47 (12–210) months. The bone cavity after curettage was filled with bone graft material (autogenous bone, 4 cases; artificial bone, 38 cases; bone cement, 1 case; allogeneic bone, 11 cases; unknown type of bone graft, 1 case). In 2 cases, both autogenous bone and artificial bone were used concurrently. In 6 cases, no replacement material was provided. Denosumab was administered preoperatively in two patients who were initially diagnosed with GCT by biopsy. A high-speed burr was used in 13 cases, not used in 28 cases, and its use is unknown in 18 cases. Thermal ablation with electrocautery was used in 19 cases, not used in 12 cases, and its use is unknown in 28 cases. Local recurrence occurred in 5 patients (5/57, 9%), but none had local recurrence after secondary surgery. No metastases were observed in any of the patients. None of the patients’ demographic data, including age, tumor size, sex, ABC-like changes on MRI, and preoperative physis status, were associated with an increased risk of local recurrence (Table 1). In the univariate analysis, no treatments or adjuvant procedures were found to be related to the risk of local recurrence. No local recurrences were observed in the two patients who underwent resection or received preoperative denosumab. The LRFS rate of all patients was 92.7% at 2 years and 88.3% at 5 years (Fig. 3A). Age < 17 years at the time of surgery was associated with an increased risk factor for local recurrence (Fig. 3B, *P* = 0.0198). Similarly, patients with an open epiphyseal line tended to experience fewer local recurrences (Fig. 3C, *P* = 0.0597). The use of a high-speed burr tended to result in fewer local recurrences, with a 100% 2-year LRFS rate in the burr group compared to 87.1% in the non-burr group (Fig. 4A, *P* = 0.4913). The 2-year LRFS rate was 88% with thermal ablation and 90.9% without thermal ablation (Fig. 4B, *P* = 0.8394). Patients who did not receive bone replacement materials experienced no local recurrences. The LRFS rate for patients who received bone replacement materials was 92.1% at 2 years and 87% at 5 years (Fig. 4C, *P* = 0.4476). The LRFS rate at 5 years was 91.4% for artificial bone, 83.3% for allogeneic bone, and 75% for autogenous bone (Fig. 4D). To eliminate the confounding effect of age on local recurrence, we conducted an analysis of adjuvant surgical procedures in cases involving patients under 17 years of age. The results remained consistent with those obtained before controlling for the effect of age (Supplementary Fig. 1). In the analysis of cases involving patients under 17 years of age, while no statistically significant difference was observed, the use of a high-speed burr showed a tendency to reduce local recurrences (Supplementary Fig. 1A, *P* = 0.4552). The postoperative function was favorable in all the patients. The MSTS score was 30 in 55 patients and 25–29 in four patients. At the last follow-up, joint degeneration was evident in 4 patients, absent in 44 patients, and unknown in 11 patients. Adjuvant surgical procedures, such as the usage of high-speed burr, ablation, or bone grafting, showed no significant impact on joint degeneration or functional impairments. Patients with recurrent chondroblastoma had higher levels of joint degeneration and disability (Table 2.).

**Table 1:** Univariate analysis of recurrence in patients with chondroblastoma

			**Recurrence**		
		**Total**	**(-)**	**( +)**	***P* value**
**Patient demographics**					
Mean age at surgery (years, range)			19 (9–42)	15 (13–16)	0.2531
Mean follow-up period (months, range)			51 (12–173)	67 (14–210)	0.7263
Mean size of tumor (cm, range)			3.3 (1–10)	3.1 (2–4.5)	0.8844
Sex	Male	42	40	2	0.1381
	Female	17	14	3	
Radiological ABC-like changes	Yes	19	18	1	0.6089
	No	22	19	3	
	Unknown	18	17	1	
Growth plate status	Open	24	20	4	0.2896
	Close	33	32	1	
	Unknown	2	2	0	
**Treatments**					
Type of Surgery	Curettage	57	52	5	> 0.999
	En bloc resection	2	2	0	
Curettage using a high-speed burr	Yes	13	12	1	0.8456
	No	26	23	3	
	Unknown	18	17	1	
Ablation	Yes	19	17	2	> 0.999
	No	12	11	1	
	Unknown	28	26	2	
Replacement material	Allograft	11	10	1	0.5331
	Autograft	4	3	1	
	Artificial bone	38	35	3	
	Bone cement	1	1	0	
	Unknown	1	1	0	
	None	6	6	0	
Denosumab	-	57	52	5	> 0.999
	+	2	2	0	

Mann–Whitney U test or Fisher's exact test

**Table 2:** Univariate analysis of joint degeneration and MSTS scores in patients with chondroblastoma

		Joint degeneration				MSTS score		
		Yes	No	Unknown	*P* value	30	25–29	*P* value
	Total	4	44	11		55	4	
Recurrence	(-)	2	42	10	0.0258	52	2	0.0326
	(+)	2	2	1		3	2	
Curettage using a high-speed burr	(-)	1	22	3	0.5552	23	3	> 0.999
	(+)	2	10	1		12	1	
Ablation	(-)	0	12	0	0.5097	12	0	0.5097
	(+)	2	17	0		17	2	
Replacement material	(-)	0	6	0	0.5692	6	0	> 0.999
	(+)	4	38	11		49	4	

Fisher’s exact test

### Cases with local recurrence

**Fig. 5:**
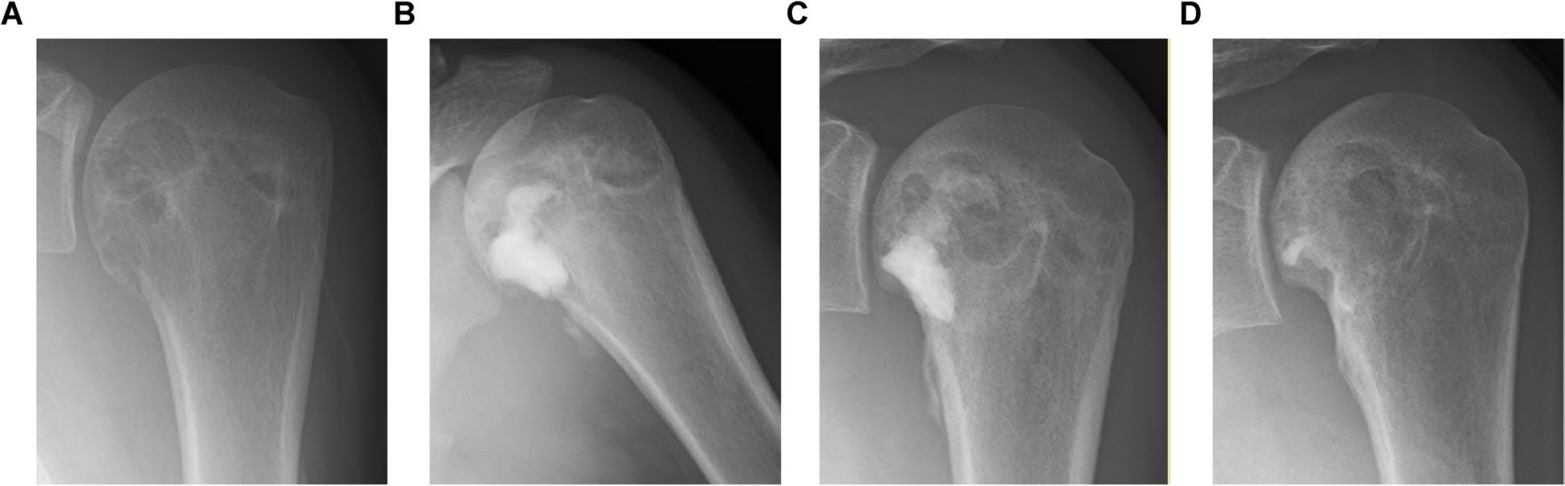
Radiographs of a chondroblastoma in the proximal humerus. Radiographs at presentation (**A**), initial surgery (**B**), time of local recurrence (**C**), and last follow-up (**D**). A 16-year-old boy presented with chondroblastoma of the proximal humerus. Curettage and ablation were performed without using high-speed burrs during the first surgical procedure. The patient underwent artificial bone grafting. Seven months after the surgery, the patient's symptoms flared, and the translucent image near the joint enlarged. The patient underwent another curettage procedure, and artificial bone grafting was performed. Seven months after the reoperation, there was no apparent local recurrence. The Musculoskeletal Tumor Society (MSTS) score was 30, although a slight degeneration was observed from the proximal humerus to the neck

**Fig. 6:**
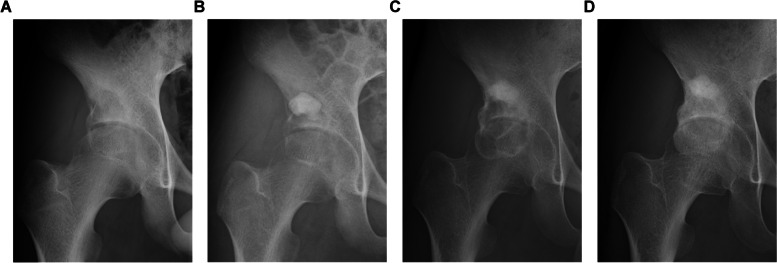
Radiographs of a chondroblastoma in the acetabulum. Radiographs at presentation (**A**), initial surgery (**B**), time of local recurrence (**C**), and last follow-up (**D**). Chondroblastoma in the acetabulum of a 15-year-old boy. The initial surgery performed was curettage. Thermal ablation was performed without using a high-speed burr during the initial surgery. Subsequently, artificial bone grafting was performed. After the initial surgery, remnants of translucency near the joint were observed, raising concerns regarding inadequate curettage and artificial bone grafting near the joint. The patient's pain improved postoperatively. However, 13 months after surgery, the symptoms flared up, and the translucent image near the joint enlarged, leading to a diagnosis of local recurrence. Four months after the second surgery, no local recurrence was observed. The Musculoskeletal Tumor Society (MSTS) score was 28

Local recurrence was observed in five patients (3 males and 2 females) who underwent curettage. The age of all the patients at the time of surgery was < 17 years (Table 3). Local recurrences occurred in the proximal humerus (2 cases) and one each in the calcaneus, pelvis (acetabulum), and distal femur. Tumor sizes ranged from 2 to 4.5 cm. The median time to local recurrence was 14 (range: 7–55) months. All patients underwent secondary curettage, and no further local recurrences were observed. Two patients subsequently developed joint degeneration. Of these patients, the MSTS score was 30 in three patients and 25 and 28 in two patients, respectively. Representative radiologic images of the local recurrent cases are shown in Figs. 5A-D and 6A-D.

**Table 3:** Recurrent cases

					1st surgery (curettage)				2nd surgery (curettage)					
Case	Age at surgery	Sex	Location	Size (cm)	Replacement materials	Use of high-speed burr	Ablation	Time to recurrence (months)	Replacement materials	Use of high-speed burr	Ablation	Oncological outcome	Joint degenetation	MSTS score
1	13	M	Proximal humerus	2	Artificial bone	No	Yes	18	Artificial bone	No	No	No further LR (NED 6 months)	No	30
2	15	F	Calcaneus	4.5	Allograft	Yes	Unknown	55	Allograft	No	Unknown	No further LR (NED 12 years)	Yes	25
3	15	M	Acetabulum	4	Artificial bone	No	No	13	Allograft	Yes	No	No further LR (NED 4 months)	No	28
4	16	M	Proximal humerus	2	Artificial bone	No	Yes	7	Artificial bone	Yes	Yes	No further LR (NED 7 months)	Yes	30
5	16	F	Distal femur	3	Autograft	Unknown	Unknown	14	Bone cement	Unknown	Unknown	No further LR (NED 5 years)	Unknown	30

*LR* local recurrence, *NED* no evidence of disease

### Cases with preoperative denosumab

Two patients who were initially diagnosed with GCT by biopsy received preoperative denosumab administration. Both patients had tumors in the proximal tibia and were 18 and 21 years old, respectively. The patients underwent curettage, and no local recurrence was observed. One patient had joint degeneration; however, the MSTS score was 30 at the final follow-up (Table 4).

**Table 4:** Case descriptions with preoperative denosumab use

Case	Age at surgery	Sex	Location	Size (cm)	Replacement materials	Use of high-speed burr	Ablation	Preoperative Denosumab (cycles)	Oncological outcome	Joint degenetation	MSTS score
1	18	M	Proximal tibia	3	Artificial bone	No	Unknown	4	No further LR (NED 48 months)	No	30
2	21	M	Proximal tibia	3.3	Allograft	Yes	Yes	7	No further LR (NED 57 months)	Yes	30

## Discussion

The demographic trends observed in our study align with previous studies, including the predominance of younger patients and frequent involvement of long bone epiphyses.

Curettage is frequently performed in cases with chondroblastoma of the long bones [9, 20, 22, 29]. Conversely, resection is chosen in cases involving flat bones such as the pelvis and ribs [19, 30]. In our study, 57 of the 59 cases were treated with curettage, while resection was performed in two cases involving the pelvis.

Previous studies showed that the LRFS rate 5 years after curettage was approximately 80–90% [17, 23] and that younger patients are at an increased risk of local recurrence [18, 20]. In general, our results were consistent with these findings. In patients with chondroblastoma, local recurrence occurs most frequently in the proximal humerus [6, 9, 18, 20, 21], proximal femur [8, 18, 24], pelvis [17, 18, 24], and other sites close to the trunk [7]. This was also consistent in our cases with local recurrence. The difficulty in performing curettage may be due to factors such as the inability to use a tourniquet in areas prone to local recurrence and the deep anatomical location of these sites, which complicates surgical access challenging and requires special techniques to achieve adequate visualization. Other potential risk factors for local recurrence may include male predilection [20], ABC-like changes [18, 20], and large tumor size [7]; however, none of these were identified as risk factors for local recurrence in our cases. Previous studies have shown a link between bone maturation and surgical techniques, which explains why younger patients are more susceptible to local recurrence. According to Suneja et al., less extensive curettage may be performed in younger patients to avoid potential damage to the growth plate [20]. Considering that skeletal maturation is achieved at 16 and 15 years of age in males and females in the Japanese population, respectively, this may explain why patients aged < 17 years had a high local recurrence rate [18]. As reported previously, we observed a trend toward higher local recurrence in the group with an open epiphyseal line. Therefore, we believe that the surgeon's attention to joint damage and insufficient curettage in younger patients may have significantly influenced local recurrence. The thoroughness of curettage is considered to be strongly associated with the low postoperative local recurrence rate.

Several adjuvant surgical procedures are effective in reducing chondroblastoma recurrence. A previous report stated that thorough curettage using a high-speed burr rather than an adjuvant was the cornerstone for adequate treatment [9]. Conversely, some reports suggest that the use of a high-speed burr and surgical techniques have no impact on the postoperative local recurrence of chondroblastoma [6, 17]. We observed a trend toward fewer local recurrences in cases where the high-speed burr was used. The use of a high-speed burr likely enables more thorough curettage, potentially lead to a lower local recurrence rate. Radiofrequency ablation (RFA), another representative therapy, is a safe and effective technique for treating chondroblastoma, with a local recurrence rate of approximately 10% [31, 32]. According to a meta-analysis of studies on the treatment of chondroblastoma with RFA, pain relief was achieved in all 97 cases included. This treatment was particularly effective for small lesions measuring 2.5 cm or less, with a reported local recurrence rate of 5.1% in this analysis [33]. In several cases of chondroblastoma, we administered adjuvant ablation therapy using electrocautery instead of radiofrequency; however, we were unable to ascertain whether this would diminish the risk of local recurrence. Regarding bone replacement materials, Turcotte et al. reported that the addition of bone graft after curettage appeared to reduce the recurrence rate [7]. In contrast to the findings of this previous report, our study revealed that the group that did not receive bone replacement material experienced no local recurrences. The absence of local recurrence in this group may be attributed to several factors, such as smaller tumors in many patients not necessitating bone grafts and or the age of the patients. In the group that underwent bone grafting, the 5-year LRFS rate was higher for artificial bone, followed by allogeneic bone and autologous bone. Although definitive conclusions are challenging due to the limited number of cases, our study suggests a trend: the lower the biocompatibility of the bone replacement material, the fewer local recurrences observed.

Another novel adjuvant is denosumab, an anti-RANKL antibody, which is used in the treatment of adults with osteoporosis [34, 35], bone metastases [36, 37], and GCT [38–40]. The effectiveness of denosumab in chondroblastoma, a giant cell lesion akin to a GCT, is yet to be demonstrated. Recent findings indicate a high frequency of RANKL expression in chondroblastoma [28], suggesting that denosumab may be effective for its treatment. Moreover, there have been reports on the efficacy of denosumab in the local control of lung metastases [2] and trunk bones [26, 27]. When using denosumab, chondroblastoma is more frequently diagnosed in children, which should be considered. Essential safety concerns related to bone turnover rebound and mineral homeostasis affect the use of denosumab in children. Therefore, further research is needed [41]. Notably, denosumab is known to cause osteosclerosis of the epiphyseal line in children [42]. A narrative review of denosumab in pediatric bone disorders, including children with ABC and GCT, revealed no adverse effects on bone growth or development [43]. The main adverse effects included hypocalcemia during denosumab treatment and rebound hypercalcemia after discontinuation [44]. In our study, two patients underwent denosumab treatment, which yielded relatively favorable results. However, its effects on local recurrence and functional outcomes remain unclear. The use of denosumab as adjuvant therapy for chondroblastoma poses a question for future studies.

In surgeries for chondroblastomas, which are predominantly located on the epiphysis of long bones, intraoperative manipulation near the joint may result in damage to the epiphyseal line and early onset of osteoarthritis [23]. Therefore, pursuing a conservative treatment approach is advisable to preserve joint function as much as possible. Arthroplasty in proximal femur cases has also been reported [29, 45]. Farfalli et al. reported that the joint survival was 90% at 5 years and 74% at 10 years [29]. Functional impairment and joint deformity are considered significant issues following surgical treatment of chondroblastoma. In our study, no arthroplasty was performed in any patient. However, the possibility of gradual development of joint deformities over an extended period is a subject for future investigation.

Numerous reports have suggested that the MSTS score is excellent during the postoperative period [6, 20, 24]. Suneja et al. reported that meticulous intralesional curettage alone achieved low rates of local recurrence and excellent long-term outcomes [20]. Similarly, our study's results were excellent from both oncological and functional standpoints, indicating that the current treatment methods are sufficient. In the current study, local recurrent cases had worse function and joint degeneration. This could be attributed to the duration of the follow-up period or the impact of multiple surgeries on functional disability or joint degeneration in local recurrent cases, as these factors were assessed during the final follow-up.

Local recurrences often occur within 1 year [21, 25]; however, there are cases in which local recurrence is confirmed after an extended period [9, 17, 18]. This may be because some patients may experience minor postoperative symptoms and imaging findings, potentially leading to the oversight of local recurrence, even if they are latent. In our study, local recurrence typically occurred within a year of surgery, with one outlier presenting at 55 months. These findings underscore the importance of extended follow-up in detecting late recurrences.

This study indicated that younger patients have a higher risk of local recurrence, and those experiencing local recurrence exhibit mild postoperative dysfunction. Despite potential technical challenges, conducting thorough curettage of the epiphysis while exercising caution to avoid damage to the epiphyseal line or articular surface is imperative. As frequently practiced in many of our cases, employing a high-speed burr with a bone replacement material such as artificial bone may offer a viable approach to mitigate the risk of local recurrence while preserving the joint. Additionally, it is essential to conduct proper imaging evaluations and follow-ups to prevent oversight and untreated persistence of local recurrence over an extended period. Although chondroblastoma is classified as a benign tumor, local recurrence and joint deformities may become apparent over time, necessitating vigilant long-term follow-up. Early detection of local recurrence may minimize joint deformities and functional impairments caused by lesions and surgical interventions.

This study had some limitations. First, this retrospective study had limited cases treated by several surgeons and mixed data from multiple centers over a long period of time. However, all the participants in the study were treated by orthopedic surgeons within the same team of musculoskeletal tumor services in the Department of Orthopaedic Surgery of Keio University School of Medicine. Although multiple surgeons performed the surgeries, the surgical procedures were traditionally shared within the service, so there should be no major differences between hospitals or areas. Second, there were several cases in which detailed information on adjuvant surgical procedures (use of high-speed burrs or ablation by electrocautery) at the time of surgery was unavailable. Third, H3K36M immunostaining was used to confirm the diagnosis in recent cases; it could not be used in past cases. However, all diagnoses of chondroblastoma were reached through the consensus of experienced pathologists, radiologists, and musculoskeletal oncologists. Despite its limitations, we believe this study is worthy of reporting, owing to the paucity of large-scale reports that incorporate an accurate histological diagnosis of chondroblastoma using H3K36M immunostaining. Finally, postoperative follow-up methods have not been standardized, and the intervals between postoperative visits and imaging evaluation methods differ between patients and physicians. Therefore, the evaluation of the time of local recurrence and metastasis may not be accurate.

## Conclusions

The characteristics of chondroblastoma and factors contributing to local recurrence after surgery were retrospectively investigated. Good oncological and functional outcomes were achieved. Patients with chondroblastoma occurring at a younger age have a higher local recurrence rate after surgery. Patients who experienced local recurrence had slightly worse postoperative function compared to those who did not experience local recurrence. To reduce the risk of local recurrence, the use of a high-speed burr may be advisable. Furthermore, if bone grafting is deemed necessary, materials with low biocompatibility, including artificial bone, should be chosen. Further exploration of various adjuvant therapies, including denosumab, is warranted to mitigate the risk of local recurrence and preserve function.

## Supplementary Information

Supplementary Material 1: Supplementary Fig. 1. LRFS curves of the groupsdefined based on various adjuvant surgical procedures. LRFS curves of the groups defined by the usage of high-speed burr, ablation, bone replacement materials, and the type of bone replacement materials

## Data Availability

The dataset used in this study is not publicly available, but can be made available upon reasonable request.

## Abbreviations

ABC: Aneurysmal bone cyst
GCT: Giant cell tumor of bone
LRFS: Local recurrence-free survival
MRI: Magnetic resonance imaging
MSTS: Musculoskeletal Tumor Society
OA: Osteoarthritis
RANKL: Receptor activator of nuclear factor-kappa B ligand
RFA: Radiofrequency ablation
