# Supplementary figures and images for: Clinical characteristics and outcomes of patients with chondroblastoma undergoing surgery with various adjuvant procedures: a retrospective study of 59 cases
Source: BMC Surg. 2025 Jan 24;25:40. doi: 10.1186/s12893-025-02782-3 (PMC11761176; doi:10.1186/s12893-025-02782-3)

## Slide 1
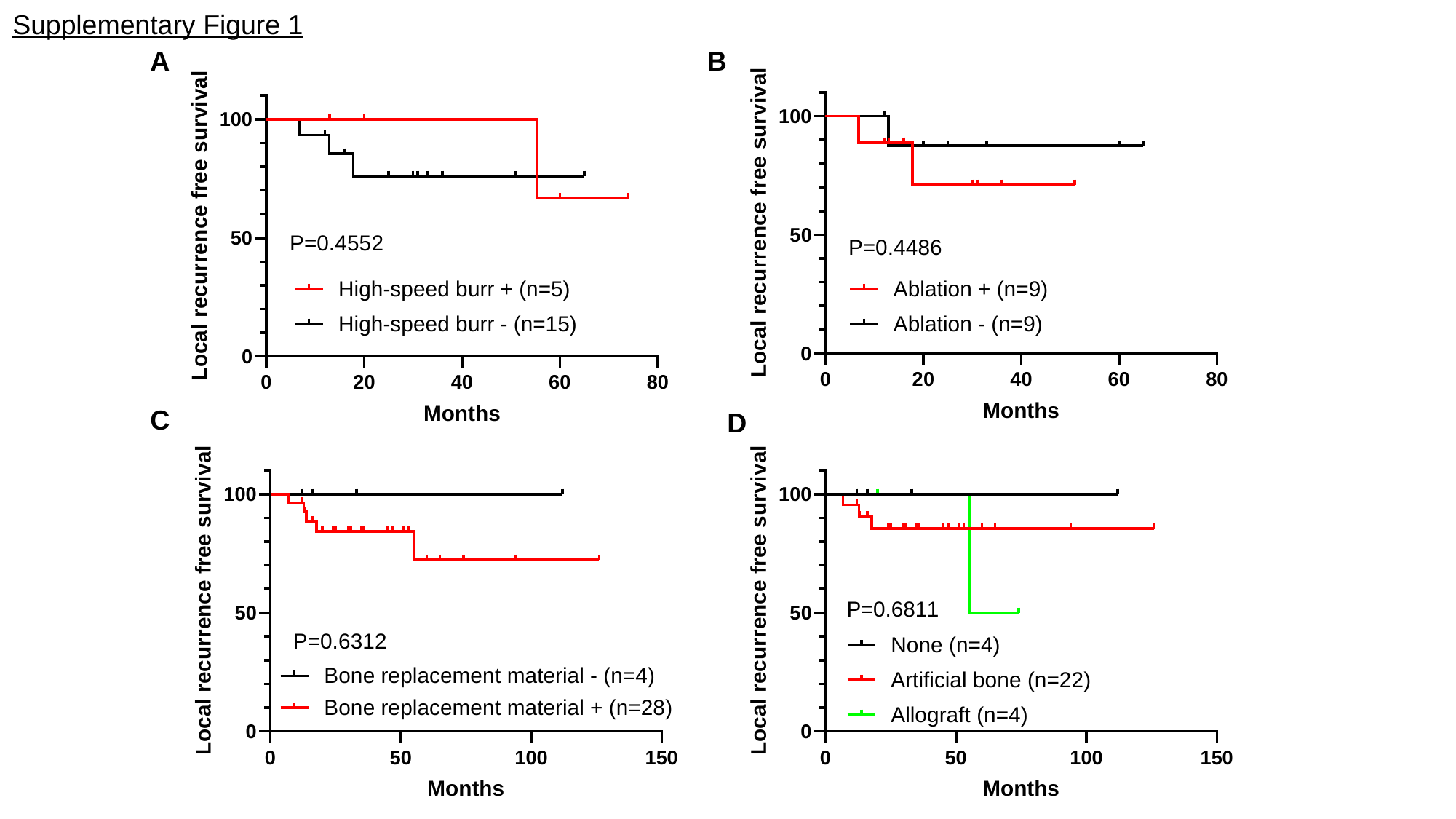

Supplementary Figure 1
A
B
C
D

Supplement: Supplementary file 1 — Supplementary Material 1: Supplementary Fig. 1. LRFS curves of the groupsdefined based on various adjuvant surgical procedures. LRFS curves of the groups defined by the usage of high-speed burr, ablation, bone replacement materials, and the type of bone replacement materials [file 12893_2025_2782_MOESM1_ESM.pptx]
